# 

**Published:** 1855-03

**Authors:** 


### CONTENTS

| | PAGE |
|---|---|
| ORIGINAL COMMUNICATIONS:— | |
| ART. I.—A Lecture on the Effects of Alcoholic Drinks on the Human System, and the Duties of Medical Men in Relation thereto. By N. S. Davis, M.D., | 97 |
| ART. II.—Amputation | 124 |
| ART. III.—Disease of the Heart. By R. S. L., | 125 |
| BOOK NOTICES. | |
| Diseases and Injuries of Seamen, &c., | 127 |
| Transactions of the American Medical Association, Vol. VII., | 131 |
| EDITORIAL: | |
| Statistics of Mortality, | 137 |
| Prof. Brainard's Prize Essay, | 139 |
| Rush Medical College Commencement, | 141 |

### RECEIPTS FROM JAN. 27 UP TO FEB. 27, 1855.

**ILLINOIS.**

| | | | |
|---|---|---|---|
| Dr E S Cooper | $4 00 | Dr W E Wilson | $2 00 |
| J W H Davis | 2 00 | L A Engle | 2 00 |
| A L McArthur | 2 00 | R S Halleek | 2 00 |
| C G Strobecker | 2 00 | J C McMurtry | 2 00 |
| T R May | 2 00 | Elias Wenger | 2 00 |
| S Cummings | 2 00 | L Hale | 1 00 |
| J A Faris | 2 00 | J W Trabue | 2 00 |
| Dr J Newton | $2 00 | Dr R M Gilkeson | $2 00 |
| B F Hutson | 2 00 | Wm Manson | 2 00 |

**INDIANA.**

| | | | |
|---|---|---|---|
| Drs Constant and Walker | 5 00 | Dr J W Newlin | 9 00 |
| | | M R Chadwick | 2 00 |

**WISCONSIN.**

| | |
|---|---|
| Dr H Warne | $2 00 |
| Edward Hopkins | 2 00 |

**MICHIGAN.**

| | |
|---|---|
| Dr James M Higby | 2 00 |
| A De Armond | 2 00 |

| | |
|---|---|
| Dr G N B Elliott, Iowa, | 2 00 |
| G F Beekner, Missouri | 2 00 |
| R L Brown, Pennsylvania, | 2 00 |

